# Chronic Headache Education and Self-management Study (CHESS) – a mixed method feasibility study to inform the design of a randomised controlled trial

**DOI:** 10.1186/s12874-019-0672-5

**Published:** 2019-02-11

**Authors:** Kimberley White, Rachel Potter, Shilpa Patel, Vivien P. Nichols, Kirstie L. Haywood, Siew Wan Hee, Dipesh Mistry, Dawn Carnes, Stephanie J. C. Taylor, Martin Underwood, Manjit S. Matharu, on behalf of the CHESS team

**Affiliations:** 1 Warwick Clinical Trials Unit, Warwick Medical School, University of Warwick, Coventry, CV4 7AL UK; 2 Division of Health Sciences, Warwick Medical School, University of Warwick, Coventry, CV4 7A UK; 3 Faculty of Health, University of Applied Sciences, Fribourg, Western Switzerland Switzerland; 4 Centre for Primary Care and Public Health, Blizard Institute Barts and The London School of Medicine and Dentistry, Queen Mary University of London, London, UK; 5 Headache Group, UCL Institute of Neurology and The National Hospital for Neurology and Neurosurgery, Queen Square, London, WC1N 3BG UK

**Keywords:** Chronic headache, Feasibility study, Self-management, Recruitment, Outcome measures, Primary care

## Abstract

**Background:**

Self-management support programmes are effective in a range of chronic conditions however there is limited evidence for their use in the treatment of chronic headaches. The aim of this study was to test the feasibility of four key aspects of a planned, future evaluative trial of a new education and self-management intervention for people with chronic headache: 1) recruiting people with chronic headache from primary care; 2) a telephone interview for the classification of chronic headaches; 3) the education and self-management intervention itself; and 4) the most appropriate patient reported outcomes (PROMS).

**Methods:**

Participants were identified and recruited from general practices in the West Midlands of the UK. We developed a nurse-led chronic headache classification interview and assessed agreement with an interview with headache specialists. We developed and tested a group based education and self-management intervention to assess training and delivery receipt using observation, facilitator, and participant feedback. We explored the acceptability and relevance of PROMs using postal questionnaires, interviews and a smartphone app.

**Results:**

Fourteen practices took part in the study and participant recruitment equated to 1.0/1000 registered patients. Challenges to recruitment were identified. We did 107 paired headache classification interviews. The level of agreement between nurse and doctor interviews was very good. We piloted the intervention in four groups with 18 participants. Qualitative feedback from participants and facilitators helped refine the intervention including shortening the overall intervention and increasing the facilitator training time. Participants completed 131 baseline questionnaires, measurement data quality, reliability and validity for headache-specific and generic measures was acceptable.

**Conclusion:**

This study indicated that recruiting people with chronic headache from primary care is feasible but challenging, our headache classification interview is fit for purpose, our study intervention is viable, and that our choice of outcome measures is acceptable to participants in a future randomised controlled trial (RCT).

**Trial registration:**

ISRCTN, ISRCTN79708100. Registered 16th December 2015, http://www.isrctn.com/ISRCTN79708100

## Background

Self-management support programmes have an established place in the management of a range of chronic diseases [1], however evidence for self-management programmes for use in chronic headaches disorders is currently limited [2]. The National Institute for Health Research (NIHR) funded a programme of work (RP-PG-1212-20,018) to develop, and test, a non-pharmacological approach for chronic headache using education and self-management. NIHR programme grants fund research for conditions that cause substantial disease burden and usually consist of ‘an interrelated group of high quality projects focused on a coherent theme, requiring multidisciplinary approaches, including clinical, health economics, statistics, qualitative and behavioural sciences, to ensure that research objectives can be met’. [3] Here we report the findings from a feasibility study we completed as part of our programme of work in preparation for a randomised controlled trial (RCT) evaluating the effectiveness of the intervention.

We wanted to test the feasibility of four key aspects prior the planned trial. Firstly, we wanted to test the feasibility of recruiting people with chronic headache from primary care and estimate the population base needed to recruit enough participants for the trial. Nearly a fifth of trials in 2011 were terminated for not meeting sufficient recruitment targets, and therefore unable to answer their research questions meaningfully [4].

Secondly, we needed to be able to classify common chronic headaches in participants identified from primary care. Specifically we wanted to test the feasibility of using a telephone classification interview that can be used by a non-headache specialist to classify the common chronic headache disorders: chronic migraine, chronic tension type headache (TTH) and medication overuse headache (MOH). Many people with chronic headache disorders do not have an accurate diagnosis and receive inappropriate treatment of their headaches [5]. We wanted the classification interview to allow classification of headache type for both reporting and analysis purposes and to be used as part of the study intervention to allow targeted, individualised, treatment and advice. A systematic review failed to identify a simple classification tool fit for our purpose, we therefore needed to develop and validate a tool which can be used by a non-headache specialist to classify common chronic headache disorders [6].

Thirdly, we wanted to test the feasibility of developing and delivering the education and self-management support intervention for the management of common chronic headache disorders and examine the acceptability of the intervention to participants. Evaluations of complex interventions can be undermined by problems of acceptability, compliance and delivery of interventions [7].

Finally, we wanted to test the quality, acceptability and appropriateness of patient reported outcome measures (PROMs) for the trial. The selection of appropriate outcomes is crucial to the design of a trial and outcomes need to be relevant to people with chronic headaches [8]. A systematic review of the quality and acceptability of patient-reported outcome measure highlighted the paucity of good quality PROM evaluations in this population and the limited focus on measurement relevance and acceptability to end-users, that is, people with headache [9]. We therefore wanted to understand which outcomes are important and relevant to people with chronic headache, a population who are often young adults with work and family commitments. Additionally, electronic diaries have shown to be acceptable to participants and may have the advantage of reducing recall effects [10, 11]; we wanted to test the feasibility of using a smartphone app to collect weekly data on headache frequency, duration and severity.

## Methods

**Fig. 1:**
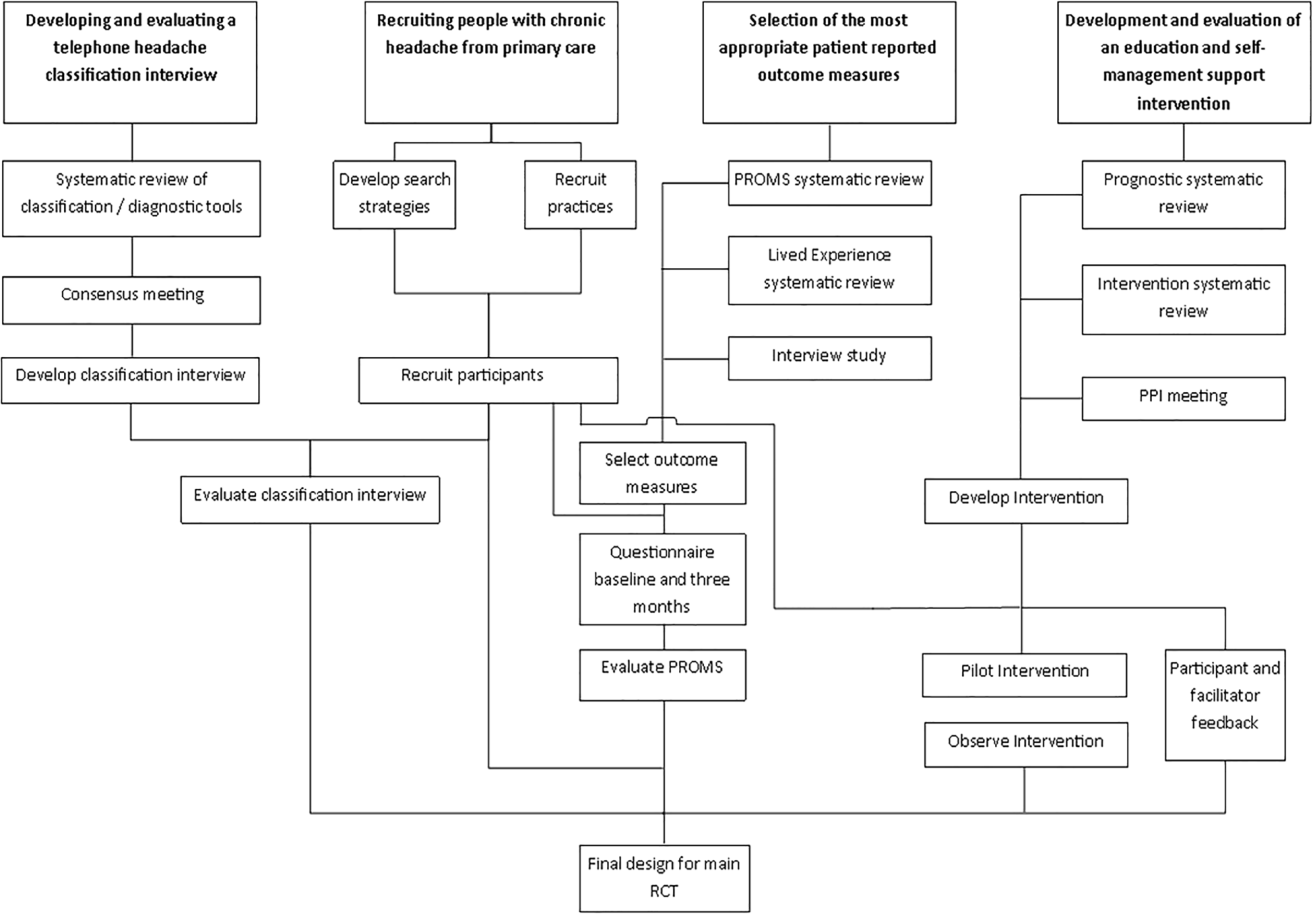
Components of the Feasibility Study

This feasibility study was designed to determine what can be done, what should be done and how it can be done well for a future RCT [12]. It was a mixed method study to test and evaluate the feasibility of a newly developed education and self-management intervention for chronic headaches, future trial recruitment methods and the most appropriate outcome measures. It included, in addition, an embedded reliability study for the classification of headaches disorders, reported in more detail elsewhere [13]. The components of the feasibility study are shown in Fig. 1. We did not conduct a full pilot trial but chose to test the feasibility of four crucial components of the main randomised controlled trial due to the complexity and importance of each of these components. The study ran from January 2016 to April 2017.

### Patient and public involvement

Patient and public involvement (PPI) was built into the key stages of the feasibility study to ensure that the research focused on issues that were important and relevant to patients and the public [14]. At the start of the study we established a lay advisory group of people with chronic headache to work with collaboratively. We identified members of the group from Universities/User Teaching and Research Action Partnership (UNTRAP) at the University of Warwick and sent out an advert to our three partner headache groups: Migraine Trust, Migraine Action and National Migraine Centre (In 2018 Migraine Action merged with Migraine Trust). The CHESS Lay Advisory Group specifically supported our application for ethical approval for the study, development of the headache classification interview, development of the study intervention and the choice of patient reported outcome measures.

### Feasibility of recruiting people with chronic headache from primary care

The aim of this part of the study was to test the feasibility of our recruitment procedures and recruit a sample of participants to test the telephone headache classification interview, to pilot the education and self-management intervention, and to test the feasibility and the outcomes measures.

#### Setting

We aimed to recruit patients with chronic headache registered with general (family) practices in the West Midlands region of the UK. We ran the study in three clinical commissioning groups (CCGs) in the West Midlands which cover urban, small town and semi-rural areas with varying levels of deprivation and ethnic diversity. We initially ran the study in five Clinical Research Network (CRN) West Midlands South ‘host practices’ with extensive research experience. Subsequently we purposively selected additional practices to maximise diversity and to fill groups for the pilot intervention. We sought feedback via email using a short structured questionnaire from a small sample of General Practitioners (GPs) from the participating practices to explore their experience of taking part in the study.

#### Participants

The eligibility criteria for the feasibility study were:

- *Inclusion Criteria:*
  1. Aged ≥18 years with chronic headache; defined as headache for 15 or more days per month for at least 3 months.
  2. Able and willing to comply with the study procedures and provide written consent.
  3. Fluent in written and spoken English.
- *Exclusion Criteria*:
  1. Has an underlying serious psychiatric or psychological disorder that precludes participation in the group intervention.
  2. Known secondary cause of headache other than medication overuse headache; e.g.: primary or secondary brain tumour.
  3. No access to a telephone.
  4. Currently participating in another clinical trial (with an unregistered medicinal product), or less than 90 days have passed since completing participation in such a trial.

The recruitment process to identify people for the study involved a standardised electronic search for general practice databases using Read-codes [15]. Initial scoping work indicated this standard clinical terminology system for coding chronic headache was rarely used, we therefore devised a search strategy to identify patients aged ≥18 years who had consulted with headache (migraine, TTH and medication overuse headache) or had been prescribed migraine specific drugs (i.e. triptans, pizotifen) in the preceding 12 months. GPs then screened the list for patients it would be inappropriate to approach e.g. poorly controlled serious mental illness, terminal illness, or known secondary causes of headache other than medication overuse headache.

Potentially eligible patients were invited to participate in the study by a letter from their GP which also included a patient information leaflet informing them about the study. We also designed a study poster for display in patient waiting areas. People interested in the study were invited to contact the study team and asked the following questions to confirm eligibility:

1. On average how many days in the month do you get headaches?
2. How long have you been having your headaches this frequently for?
3. Has this been for at least the last 3 months?
4. Are you currently taking part in a drug trial?

Patients who met the eligibility criteria were informed that they would be asked to complete two telephone headache classification calls (one by a nurse the second by a headache specialist doctor) and that they may be invited to attend the education and self-management programme and/or take part in the interview study. Potential participants also had the opportunity to have any questions answered regarding the study.

Baseline packs were sent to people who were eligible and interested in the study, they included a consent form, a baseline questionnaire and a freepost return envelope. If necessary, a reminder pack was sent after 2 weeks. All participants were asked to provide written consent to complete postal questionnaires, a Smartphone App and the two telephone classification interviews. Study entry was marked by receipt of the signed consent form.

#### Sample size

For the suite of work in the feasibility study we initially sought to recruit 170 people with chronic headaches from primary care. The driver for this sample size was to have sufficient data to allow us to assess the inter-rater reliability of the telephone classification interview when done by two raters; namely a nurse, and a doctor experienced in headache management. We assumed level of agreement to be 0.8, a substantial agreement [16]. The initial sample size was based on measuring the level of agreement for the classification of migraine (yes/no), TTH (yes/no) and MOH (yes/no). Following our systematic review of diagnostic tools and our classification consensus meeting, the outcomes from the classification changed to measuring the level of agreement in the classification of definite chronic migraine, probable chronic migraine and chronic TTH as well as presence or absence of MOH as a nominal scale. As the analyses changed from three pairwise comparisons to two pairwise comparisons, the multiplicity adjustment also changed hence giving a revised sample size target of 153 paired interviews which was approved by the programme steering committee and the funder [17].

An initial pilot search suggested that around 30/1000 people registered with a GP consult for headaches (acute, episodic or chronic) annually. Assuming that a third of these consulters had chronic headaches and a quarter of these joined the feasibility study recruitment rate would be 2.5/1000 or 8.3% of those identified as consulting with headaches. Based on an average practice population of 7000 we estimated we needed 6–10 practices with a combined list size of 64,000 people to recruit our sample.

### Feasibility of a telephone classification interview to classify common headache disorders

We developed a telephone headache classification interview for use by a non-headache specialist to classify chronic headache types for reporting and analysis purposes and that could also be used as part of the study intervention to allow targeted treatment and advice. In brief, we did a systematic literature review to identify any existing tools used to classify or diagnose different headache types which was presented to delegates at a headache classification consensus conference attended by headache specialists and people with chronic headache [6]. At the consensus conference delegates agreed what were the important questions to include in the classification interview. The classification interview was not intended to have a rigid interview structure or set questions, instead the person conducting the interview was encouraged to use a logic model to inform their clinical reasoning and decision-making.

We aimed to test the feasibility of training nurses to use the classification interview to classify chronic headache disorders and test the reliability of the tool. To validate the classification interview we trained six nurses, all non-headache experts, to conduct the interviews. The training included a one-day workshop delivered by a neurologist specialised in headache plus time with a member of the study team to practice classification interviews using mock scenarios and a training manual.

Participants from the feasibility study were interviewed first by the nurse and later by a doctor from the National Migraine Centre. The doctor classification was the assumed ‘gold standard’. Participants were classified into: definite chronic migraine, probable chronic migraine or chronic TTH (with or without medication overuse) or ‘other’ headache type (other chronic primary headache or suspected secondary headache). We measured level of agreement between the classifications by nurses and doctors by using simple kappa statistics and prevalence-adjusted bias-adjusted kappa (PABAK).

The development and evaluation of the telephone headache classification interview is described in detail elsewhere [13].

### Feasibility and acceptability of the education and self-management support intervention for chronic headache

We developed the education and self-management intervention using the Medical Research Council (MRC) framework for complex interventions [7]. Development was informed by three systematic reviews 1.prognostic factors in chronic headache [18], 2. education and self-management interventions for chronic headache [19] and 3. the lived experiences of chronic headache. We drew from the experience of a previously tested self-management intervention for chronic pain [20] and we did qualitative interviews with people with chronic headache to inform the intervention design. The qualitative interviews were with members of the charity Migraine Action to gain their views on what was important to include in the education and self-management intervention. We held a collaborative intervention design meeting, attended by headache specialist clinicians, headache charity representatives, lay people with chronic headache, psychologists, and researchers.

The education and self-management intervention was intended to be delivered in a group format (8–10 per group) facilitated by a nurse and a lay person (with chronic headache). Topics included in the intervention were: understanding headache mechanisms, medication management, mood and headache, recognising unhelpful thought patterns and behaviours, stress management, sleep management, communication and mindfulness. The two and a half day programme used a range of methods including: group discussions, sharing narratives and experiences, problem solving, watching an educational DVD, role play and taster sessions. This was followed by a one to one consultation with a nurse to classify their headache type and discuss medication, lifestyle factors and goal setting, and up to 8 weeks of telephone support.

The development of the education and self-management interventions is described in detail elsewhere [21].

We aimed to test the feasibility of the new intervention by running four groups each with up to 10 participants in community settings. We approached people who lived within easy travelling distance of proposed groups; participants provided written consent to attend the group intervention. We wanted to recruit and train two lay people and three nurses to deliver the intervention. The acceptability of the intervention was explored by conducting qualitative interviews with the participants who attended the groups and the facilitators that delivered the groups. Thematic analysis was used to identify common themes across the different components of the intervention.

### Feasibility of the patient reported outcome measures

We proposed that our primary outcome measure for the RCT would be a headache-specific outcome measure collected by postal questionnaire. We initially did a systematic review of the quality and acceptability of patient reported outcome measures for episodic and chronic headache disorders [9], and a qualitative review of the lived experience of chronic headache [22] to understand what outcomes are important to people with chronic headache. This process supported the short-listing of both headache-specific (Migraine-Specific Questionnaire v2.1(MSQv2.1) [23] and the Headache Impact Test 6-item (HIT-6) [24] and generic measures (EuroQoL EQ-5D-5 L) [25] and Short-Form 12-item Health Status questionnaire (SF-12) [26] to include in the feasibility study. However, the migraine-specificity of the MSQv2.1 [23] made it unsuitable for use with our chronic headache population. Therefore, with permission from the developers, the target attribute of ‘migraine’ was changed to ‘headache’ and the questionnaire renamed as the ‘Chronic Headache Quality of Life Questionnaire’ (CHQLQv1.0). We evaluated both the acceptability and psychometric performance (data quality, reliability, validity) of the modified measure against the HIT-6, EQ-5D-5 L and SF-12 [24–26], providing the first evidence for the performance of the CHQLQ and HIT-6 [24] in a UK population and supporting selection for the RCT. Structured cognitive interviews were also conducted to explore the acceptability and relevance of the measures. Informed by good practice guidance, the interviews explored how responder’s made judgements when completing the PROMs, including aspects such as question comprehension, recall and ease of completion [27, 28]. The cognitive interviews and their analysis was carried out by an experienced qualitative team with expertise in this area.

#### Data collection

All participants were asked to complete postal questionnaires with the selected measures CHQLQ, HIT-6, SF-12 and EQ-5D-5 L at baseline (the point of consent) and at 2 weeks and 12 weeks after the baseline questionnaire was returned. The study team posted the questionnaire with a covering letter and a freepost return envelope. After 1 week if the questionnaire had not been received a reminder was sent and, 1 week following the reminder a telephone call would be made if the questionnaire was not received.

A smartphone application (app) compatible with IPhones, IPads and Android devices was designed by Clinvivo Ltd. for use in the study. The app asked participants to complete three simple questions regarding the frequency, severity and duration of the headaches they experienced. The questions were developed with the involvement of the CHESS Lay Advisory Group. The app requested the data to be completed weekly for up to 12 weeks and provided notification reminders for those who accepted this option. A small number of participants were approached to test the app; these were all participants who had recently agreed to take part in the study at the time the app was ready for testing.

## Results

### Feasibility of recruiting people with chronic headache from primary care

#### Practice recruitment

**Fig. 2:**
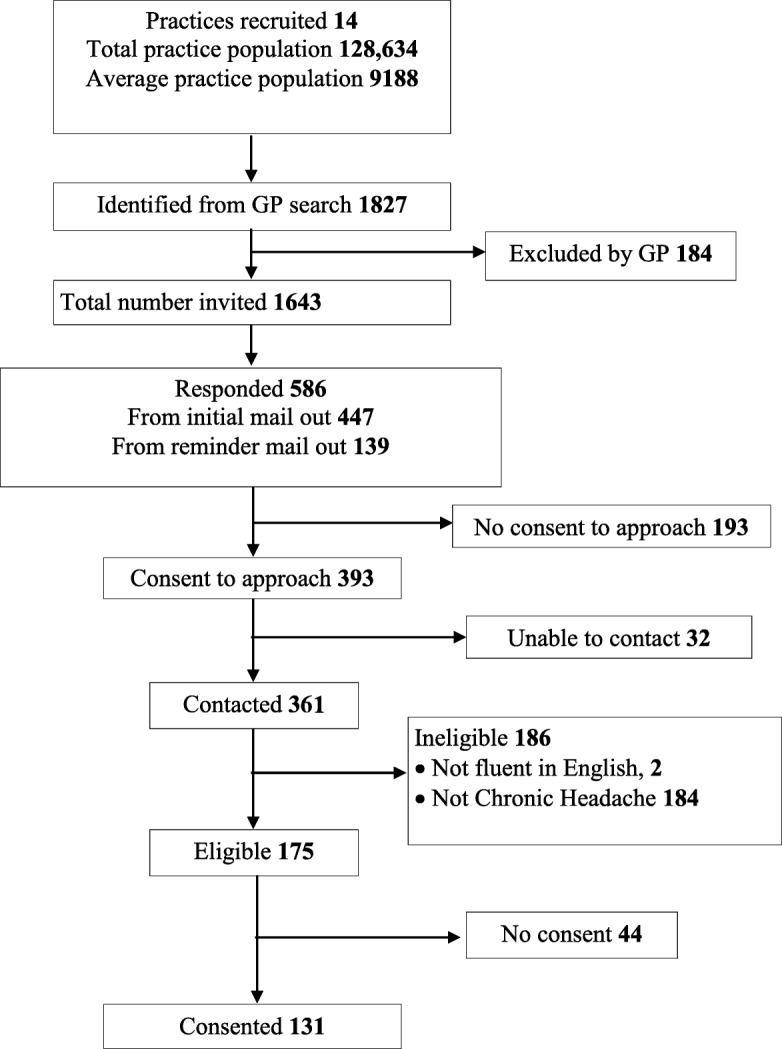
Practice and participant recruitment consort chart

We recruited 14 general practices with a combined practice population of 128,634 (range 3300 to 16,886), see Fig. 2. Feedback from the short structured email questionnaire to GPs indicated that practices were mainly interested in the study because they felt a self-management programme could potentially provide a useful alternative option for the management of patients with frequent headaches.

#### Participant recruitment

Searches of general practice data bases identified 1827 potential participants (14.2/1000 of registered patients). GPs excluded 184 (10%) of these as inappropriate to approach. The remaining 1634 (1.3% of total list size) were invited to take part in the study. We received 586 (36%) responses, of these 393 (24%) were interested in being contacted by the study team; 193 were not interested in the study. We succeeded in contacting 361/393 (92%) often after numerous attempts to get hold of people; of these potential participants 175 (48% of those contacted, 11% of those 1634 invited) were eligible. We received valid consent forms from 75% (131/175) of eligible participants (8% of those 1634 invited). Forty people failed to respond and four formally withdrew at this stage. We recruited 1.0/1000 of practice list size.

Participants mean age was 49 years (range 21–77, standard deviation, SD, 13.3). There were 108 (82%) female participants, 125 (95%) of white ethnicity and 86 (66%) in full or part-time employment. About one third (*n* = 47, 36%) left full time education between age 17 and 19, and another third (*n* = 44, 34%) left full time education after 20 years old (Table 1).

**Table 1:** Participant demographics

		Feasibility sample
		(*N* = 131)
Age (years)	N	128
	Mean (sd)	48.9 (13.3)
	Median (IQR)	49 (38.5,58)
	Missing	3
Gender	Male	21 (16%)
	Female	108 (82%)
	Missing	2 (2%)
Ethnicity	White	125 (95%)
	Black or Black British	2 (2%)
	Asian or Asian British	1 (1%)
	Mixed	1 (1%)
	Other	1 (1%)
	Missing	1 (1%)
Employment	Employed (full or part-time including self-employment)	86 (66%)
	Unemployed and looking for work	0
	At school or in full time education	2 (2%)
	Unable to work due to long term sickness	3 (2%)
	Looking after your home/family	11 (8%)
	Retired from paid work	22 (17%)
	Other	3 (2%)
	Missing	4 (3%)
Age left full time education	Did not receive formal education	0
	≤12	0
	13–16	35 (27%)
	17–19	47 (36%)
	≥20	44 (34%)
	Still in full time education	3 (2%)
	Other	1 (1%)
	Missing	1 (1%)

### Feasibility of the headache classification interview

We trained six research nurses to conduct the telephone classification interviews. Feedback from the training indicated that the nurses felt that the training workshop, opportunity to practice interviews and the training manual prepared them adequately to carry out the classification calls and that they gained confidence the more interviews they completed. Nurses and doctors from the NMC completed 111 and 108 headache classifications interviews respectively. There were 107 paired interviews. Median days between interviews was 32 (interquartile range, IQR, 21 to 48 days). Proportion of concordance of agreement between nurses’ and doctors’ interviews was 0.91, with moderate or very good agreement on PABAK agreement in main and sensitivity analyses respectively. Full details of these analyses are reported elsewhere [13].

### Feasibility of the education and self-management support intervention

**Fig. 3:**
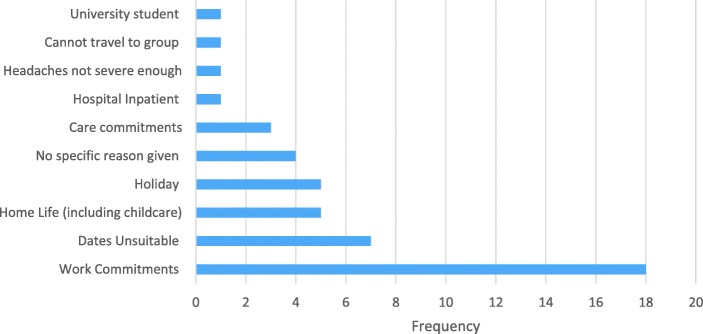
Reasons participants were unable to attend the group intervention

We approached 85 participants to pilot the education and self-management programme; we were unable to contact 12 (14%) participants and 46 (54%) participants were unable to attend, reasons included work commitments, dates being unsuitable, home life (including childcare) and holidays (Fig. 3), 27 (32%) expressed interest in attending the intervention and of these 18 (21%) provided written consent to attend a group.

We piloted the CHESS intervention in four groups and with a total of 18 participants. The attendance at groups ranged from 3 to 6 participants and 17 participants attended the one-to-one consultation with the nurse. Qualitative interviews were completed with 12 participants using topic guides to explore participants’ experience of taking part in the intervention. On the whole the groups were considered acceptable and participants found the educational and self-management components useful and interesting and found the opportunity to meet with other people with chronic headache particularly helpful. Based on participant feedback we removed the half day follow-up session because participants found the time commitment too great and we included the sessions on communication and managing setbacks at the end of day two of the programme.

Facilitators gave us feedback in a focus group or interviews with the use of topic guides, including their experiences of delivering the intervention and the training received. They reported that they did not find the two- day training adequate time to cover the delivery of the group intervention and the headache classification and medication information for the one-to-one consultations. It was also difficult for the lay facilitators to commit to delivering the intervention due to existing work and family commitments and unpredictability of their own headaches.

### Feasibility of the patient reported outcome measures

Participants completed and returned 131 baseline questionnaires; 115 (88%) and 103 (79%) questionnaires were returned at two and 12-week follow up respectively. Measurement data quality, reliability and validity for the headache-specific and generic measures was reached at acceptable standards [29, 30], supporting application of the measures with groups of patients with chronic headache. Participants in the cognitive interviews (*n* = 14) indicated items included in the CHQLQ were comprehensive in scope and particularly welcomed those referring to the emotional impact of headache, and found the measure easy to complete. The lack of recall period for the first three items of the HIT-6 was a concern. The generic measures were considered to be acceptable.

In total eight participants downloaded the Smartphone App, participants completed the app for a duration of up to 11 weeks. A telephone call was made to a selection of participants to check they were happy using the app and although participants didn’t report difficulties downloading or using the app only one participant completed all 11 weeks of data collection and only four participants completed half or more of the weeks.

## Discussion

One of the key objectives for the study was to test the feasibility of recruiting people with chronic headache from primary care and estimate the population base needed to recruit enough participants for the RCT. We successfully recruited 14 general practices to the study and feedback from GPs suggested that an invitation to participate in a randomised controlled trial is likely to be well received by general practices.

Recruitment to the study equated to around one per 1000 of the list size; this is comparable to recruitment rates from general practice for other studies of chronic pain [20, 31, 32]. It is, however, substantially less than our pre-study assumptions. The number of people with headaches across our pool of 14 practices was a little under half of that anticipated and the conversion rate of 7.1% from identification to consent was slightly less than pre-study assumptions. The highest identification rate was 18.3/1000 ranging down to 8.1/1000 (data not shown) suggesting that whilst there is great variability in coding of headache in practices our initial scoping searches were erroneous. Our conversion rate estimate was slightly optimistic and again there was a wide variability in conversion rate by practice (3.3 to 9.4%, data not shown). Consequently a wide range in recruitment rate (0.6/1000 to 1.6/1000, data not shown). This means we under-recruited against our original target and will need to recruit participants from over 100 practices for the RCT.

Overall we gained much useful information and experience from the recruitment processes for this feasibility study and we have made some important changes to our approach for the main study including allowing self-referral to the study from posters in pharmacies local to participating general practices and word of mouth media exposure. Contacting a largely young working population was challenging often requiring numerous attempts by telephone and email and a flexible approach to contacting people outside usual working hours. Only 75% of those eligible to take part returned signed consent forms despite chasing.

We had also not fully anticipated the challenges of making paired headache classification calls meaning we had data on fewer people than originally planned. Nevertheless we did obtain sufficient data to evaluate the agreement between nurse and doctor interviews. Non-headache specialist nurses were able to use our logic model to classify chronic headaches types and identify medication overuse headache and the level of agreement with interviews by doctors specialised in headache was good, giving us confidence in the classification interview in the RCT.

We successfully piloted the intervention in four groups and gained valuable feedback from participants and facilitators. The length of the group intervention was reduced by half a day because participants, found it hard to commit more time due to work and family commitments. Nurse facilitators requested more training in order to feel confident in headache classification and medication advice, and an additional day training has been added in the RCT. The group intervention was originally designed to be facilitated by a health professional and lay person with chronic headache, a model which has previously been successful for the delivery of group interventions for chronic pain [20]. Because of the unpredictability of their own headaches it was not possible for the lay facilitators to commit to the role, and in the RCT the intervention will be facilitated by a nurse and an allied health professional. Alongside the RCT we will run a process evaluation to help understand how and if the intervention works. This will include collecting data on group attendance and interviews with a sample of participants and facilitators to explore the experience of delivering and receiving the intervention to inform any future roll out of the programme.

The completion and follow up of postal questionnaires was good, and all measures were well completed by responders at all time-points. Acceptable levels of data quality, reliability and validity were found for all measures, supporting their use with groups of people and justifying selection for the RCT. Participants indicated that the modified measure the Chronic Headache Quality of Life Questionnaire was both comprehensive and comprehensible. We were able to test our Smartphone App prior to the RCT in a small sample of participants, completion rates were poorer than anticipated and strategies to improve level of completion will be implemented in the main trial.

The advice and support of PPI was integral to the intervention development and other aspects of the feasibility study and the lay advisory group will continue their contribution into the main RCT.

The findings from the feasibility study have allowed us to be confident we are selecting the right participants and have a viable intervention, and allowed us to make an informed choice about outcome measures for the RCT. The feasibility study also identified challenges in recruitment of participants with chronic headache from primary care and collecting patient reported outcome measures that we have learnt from before starting the main trail.

The CHESS RCT (ISRCTN 79708100) which commenced January 2017 will test the effectiveness and cost effectiveness of the group education and self-management intervention compared with a best usual care and a relaxation CD for people living with chronic headaches (ISRCTN 79708100).

## Conclusions

This study has demonstrated that recruiting people with chronic headache from primary care requires a large pool of patients which means recruiting many general practices and a flexible approach to contacting what is largely a young working population. We have developed and evaluated a telephone headache classification interview that can be used by a non-headache specialist to classify chronic headache disorders. We have provided essential evidence in support of a newly modified headache-specific measure, for application alongside established headache-specific and generic measures in this population. Despite our best efforts to involve lay people with chronic headache in the delivery of the intervention it was difficult due to their own person health; from a pragmatic stance the intervention was feasible when delivered by two health care practitioners.

## Abbreviations

CHESS: Chronic Headache Education and Self-management Study
CHQLQv1.0: Chronic headache quality of life questionnaire
GP: General Practitioner
HIT-6: Headache impact test 6-item
MOH: Medication overuse headache
MSQv2.1: Migraine-specific questionnaire v2.1
NIHR: National Institute for Health Research
PPI: Patient and public involvement
PROM: Patient reported outcome measure
RCT: Randomised controlled trial
SF-12: Short-form 12-item health status questionnaire
TTH: Tension type headache
UNTRAP: User teaching and research action partnership
